# Reasoning and choice in the Monty Hall Dilemma (MHD): implications for improving Bayesian reasoning

**DOI:** 10.3389/fpsyg.2015.00353

**Published:** 2015-03-31

**Authors:** Elisabet Tubau, David Aguilar-Lleyda, Eric D. Johnson

**Affiliations:** ^1^Departament de Psicologia Bàsica, Facultat de Psicologia, Universitat de Barcelona, BarcelonaSpain; ^2^Research Institute for Brain, Cognition and Behavior, University of Barcelona, BarcelonaSpain

**Keywords:** Bayesian reasoning, Monty Hall Dilemma, choice biases, cognitive illusions, reflection

## Abstract

The Monty Hall Dilemma (MHD) is a two-step decision problem involving counterintuitive conditional probabilities. The first choice is made among three equally probable options, whereas the second choice takes place after the elimination of one of the non-selected options which does not hide the prize. Differing from most Bayesian problems, statistical information in the MHD has to be inferred, either by learning outcome probabilities or by reasoning from the presented sequence of events. This often leads to suboptimal decisions and erroneous probability judgments. Specifically, decision makers commonly develop a wrong intuition that final probabilities are equally distributed, together with a preference for their first choice. Several studies have shown that repeated practice enhances sensitivity to the different reward probabilities, but does not facilitate correct Bayesian reasoning. However, modest improvements in probability judgments have been observed after guided explanations. To explain these dissociations, the present review focuses on two types of causes producing the observed biases: Emotional-based choice biases and cognitive limitations in understanding probabilistic information. Among the latter, we identify a crucial cause for the universal difficulty in overcoming the equiprobability illusion: Incomplete representation of prior and conditional probabilities. We conclude that repeated practice and/or high incentives can be effective for overcoming choice biases, but promoting an adequate partitioning of possibilities seems to be necessary for overcoming cognitive illusions and improving Bayesian reasoning.

## Introduction

Bayesian reasoning has primarily been investigated in the context of imaginary scenarios, in which participants are required to derive a posterior probability (or a posterior ratio of natural frequencies) from explicit statistical information. An exception can be found in research with the Monty Hall Dilemma (MHD), where Bayesian reasoning has been studied with both imaginary scenarios and repeated practice. Differing from typical Bayesian problems, priors and conditional probabilities in the MHD have to be inferred, either by learning reward probabilities or by reasoning from the presented sequence of events. By reviewing the main difficulties and interventions for improving either choice or probabilistic judgments in the MHD, two different causes of failures are introduced: (1) emotional-based choice biases (switch aversion and/or the endowment effect), and (2) cognitive limitations in understanding and representing probabilities. We argue that while the first cause produces illusions of control, regret, or distortions in the memory of past choice-outcome events, the second one promotes illusions of equiprobability and/or distortions in understanding the conditions of the game. The present review shows that both causes can independently and simultaneously bias choice and probabilistic judgments. Furthermore, whereas choice biases can be overcome by extended practice or by high incentives, overcoming the erroneous default intuition requires explicit instruction about the correct partitioning of probabilities. Implications for improving Bayesian reasoning are also discussed.

## Understanding the MHD: From Intuition to Bayesian Reasoning

**FIGURE 1:**
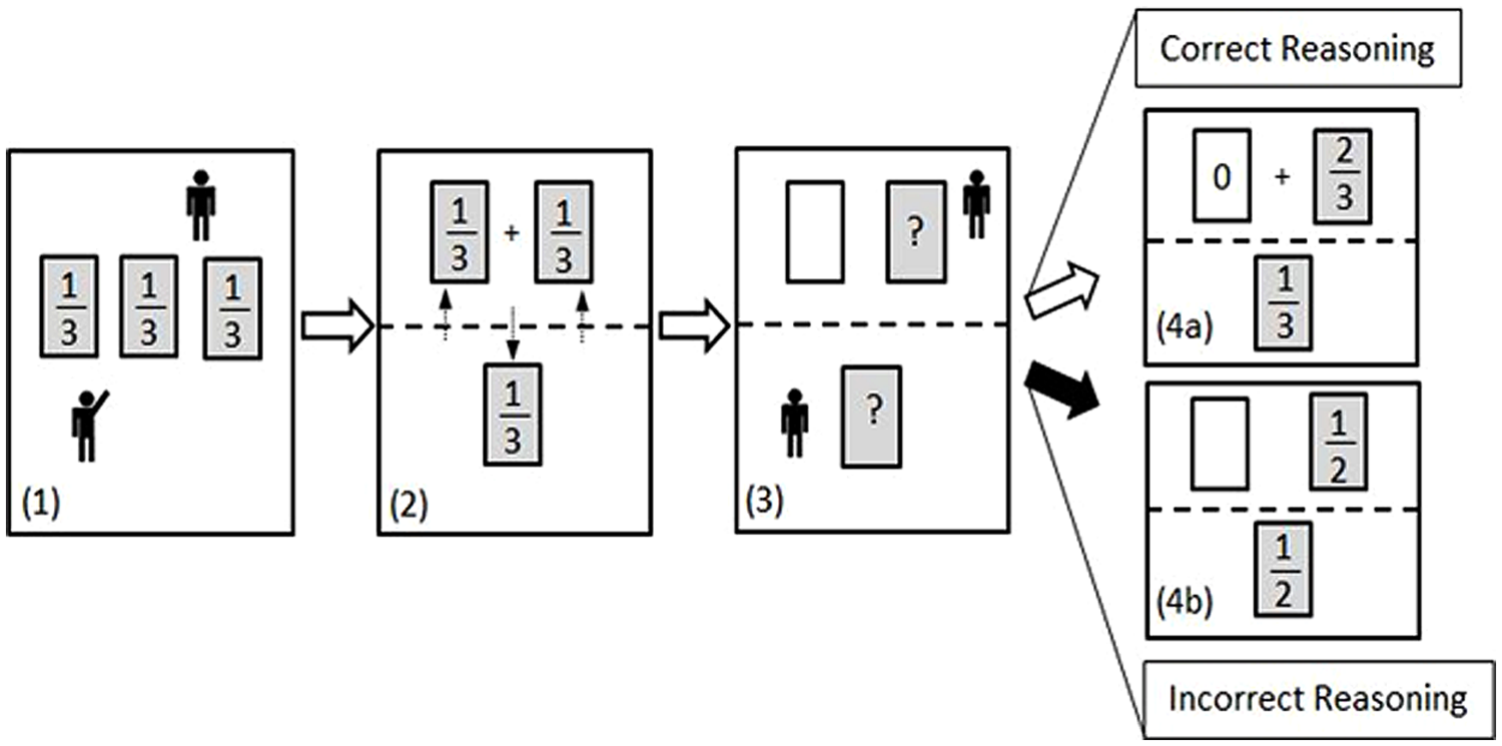
**Schematic representation of the MHD with the host (top) and player (bottom)**. (1) A player is presented three doors, each with an equal chance (1/3) of containing a prize and he chooses one of them. (2) Following the initial selection, the player now has one door with a 1/3 chance of having the prize. The host now has two doors with a total 2/3 chance of having the prize (1/3 + 1/3). (3) The host opens one of his two doors which does *not* contain the prize. The player is offered the choice to *stick* with his original selection or to *switch* to the unopened door held by the host. (4a) *Correct Reasoning*: Given that the opening of a non-rewarding door is obligatory, there still remains a 2/3 chance that the prize is on the “side” of the host, and a 1/3 chance that the prize is behind the player’s originally chosen door. (4b) *Incorrect Reasoning*: A typical cognitive error is based on the illusion of equiprobability between the two remaining doors (see further explanation in the text).

The MHD is a good example of a counterintuitive decision-making problem, considered to be “the most expressive example of *cognitive illusions* or *mental tunnels* in which even the finest and best-trained minds get trapped” (Piattelli-Palmarini, 1994; p. 161; cited by Krauss and Wang, 2003). In a first choice, a participant selects one of three possible options (i.e., doors), after being informed that only one hides a prize, and that the chances for each door are equal. Next, the host (or computer, in computer-based versions), who knows which door hides the prize, opens one non-rewarded door of the two remaining non-selected doors. The participant is then given a second, binary choice, which determines the final outcome of the game: They may either (a) stay with their initial selection [*stick*], or (b) swap their original selection for the other still closed door [*switch*]. The naïve reader would likely believe that each of the remaining two options has an equal probability of containing the prize, as often observed in the literature (i.e., Shimojo and Ichikawa, 1989; Franco-Watkins et al., 2003; Tubau and Alonso, 2003; De Neys and Verschueren, 2006; see also **Figure 1**). This common illusion has been attributed to a misapplication of the equiprobability principle (Falk, 1992; Johnson-Laird et al., 1999; Falk and Lann, 2008) due to the wrong intuition that, after the elimination of an option, all the chances have to be updated (Baratgin and Politzer, 2010). Specifically, the observation of two remaining options promotes the illusion that each of the final two options has a 50% chance of containing the prize. However, the elimination of an option (known by the host not to contain the prize) does not change the prior probability concerning the first choice. As shown in **Figure 1** and **Table 1**, the participant still has a 1/3 chance of having initially selected the prize and, therefore, in two out of three cases a decision to switch options will ultimately lead to a prize (a more formal explanation of probabilities in the MHD is introduced below).

**Table 1:** Possibilities in the MHD: the probability of each door to be opened is conditioned on both the first choice and on the location of the prize.

Prize location	First choice	Probability to open door	Remaining door (after open)	Best choice	
Door 1	**Door 1** Door 2 Door 3	*P*(each door) = 0.5 *P*(Door 3) = 1 *P*(Door 2) = 1	Door 2 or Door 3 **Door 1** **Door 1**	Stick Switch Switch	
Door 2	Door 1 **Door 2** Door 3	*P*(Door 3) = 1 *P*(each door) = 0.5 *P*(Door 1) = 1	**Door 2** Door 1 or Door 3 **Door 2**	Switch Stick Switch	
Door 3	Door 1 Door 2 **Door 3**	*P*(Door 2) = 1 *P*(Door 1) = 1 *P*(each door) = 0.5	**Door 3** **Door 3** Door 1 or Door 2	Switch Switch Stick	

*Notice that in 1 of 3 times the prize is behind the selected door and in 2 of 3 times the prize is behind the remaining door. Therefore, *P*(prize| stick) = 1/3 and *P*(prize| switch = 2/3 (in bold, location of the ace after opening one door).*

Nevertheless, the final choice is generally neither fully coherent with the actual distribution of chances, nor with the mis-application of the equiprobability principle. A large majority of participants prefer to stick with the original choice (Granberg and Brown, 1995; Krauss and Wang, 2003), a tendency that has been related to an illusion of control (Lichtenstein and Slovic, 1971; Langer, 1975; Granberg and Dorr, 1998), or to a strategy to prevent future regret, which is more strongly perceived when losing after switching (Gilovich et al., 1995; Granberg and Brown, 1995; Petrocelli and Harris, 2011). Hence, the MHD motivates two different biases that work against the optimal solution: The equiprobability illusion and emotional-based choice biases. Both types of bias are difficult to overcome because the MHD presents an additional difficulty for most people: The need to distinguish a winning probability that has to be updated (the one concerning the remaining door) from a winning probability that remains the same (the one concerning the first choice). Regarding this point, we claim that difficulties in overcoming illusions in the MHD are a consequence of a more primary cause: A biased representation of the prior probabilities. In Section “An Overlooked Failure: Incomplete Representation of Prior Probabilities” we review evidence supporting this claim.

From a Bayesian perspective, understanding the optimal solution in the MHD requires realizing that the elimination event is conditioned on both the first choice and on the location of the prize (Glymour, 2001; Burns and Wieth, 2004). Consider a scenario where the participant initially selects door 1. The conditional probability (likelihood) of eliminating, for example, door 3 after choosing door 1, depends on the hypothesis being considered (see also Falk and Lann, 2008). Specifically, given that the probability of revealing door 3 among the remaining two doors does not depend on the content of selected door 1 [*P*(D_3_| H_1_) = *P*(D_3_) = 1/2], the posterior probability of such door containing the prize, conditioned to the elimination of door 3, is the same as its prior probability of containing the prize [*P*(H_1_| D_3_) = *P*(H_1_) = 1/3]. In contrast, given that the probability of revealing door 3, conditioned to the prize being hidden in the remaining door 2, is doubled [*P*(D_3_| H_2_) = 2*P*(D_3_) = 1], the posterior probability of door 2 hiding the prize, conditioned to the opening of door 3, also doubles [*P*(H_2_| D_3_) = 2*P*(H_2_) = 2/3].

In other words, the conditions of the elimination have two main implications: (a) the winning probability for the selected door cannot change since it is conditioned to an unconditional event (it is certain that one of the non-selected doors is always null), and (b) the winning probability for the remaining door doubles, as the opening of a non-selected door is conditioned on the current location of the prize (see **Table 1**). In sum, understanding the MHD requires being able to distinguish conditional and unconditional events, or conditions in which probabilities have to be updated from conditions in which probabilities remain the same. In the following sections we review the difficulties found both in learning to choose optimally and in correct (explicit) Bayesian reasoning in the MHD in order to suggest causes and possible remediation.

## Learning to Choose Optimally in the MHD

It is a well-grounded finding that both humans and non-human animals learn to optimize choices by adapting expectancies to the probability of forthcoming outcomes (Kahneman and Tversky, 1979). In repeated two-choice tasks, an increment in the probability of an optimal choice tends to follow the *matching law* (Herrnstein, 2000). Specifically, a matching between choice and reward probabilities is commonly observed, which is considered to be a consequence of a default adaptive strategy (West and Stanovich, 2003; Koehler and James, 2010). Nevertheless, sequential decision making tasks which include dependencies between choices can produce higher learning variability, and can lead to choices which deviate substantially from programmed reward probabilities (Herbranson and Wang, 2014).

Optimal choice in these more complex scenarios can be seen as arising from a Bayesian inference; that is, the probability of the outcome can be computed by combining its prior probability and the likelihood of the new observation. Alternatively, by repeating the decisional task, optimal choice preference can also develop through learning of either the most often rewarded final choice (i.e., switch in the MHD), or of the specific sequence of choices associated with the highest reward probability (e.g., “choose the leftmost option in the three-choice scenario, then switch in the two-choice decision”). The latter seems to explain pigeons’ tendency to choose more optimally than humans in analogous MHD tasks (Herbranson and Schroeder, 2010; but see Mazur and Kahlbaugh, 2012 for similar results between species). In the case of humans, is repeated practice really useful for learning to choose optimally in the MHD? Furthermore, is this learning useful for improving correct Bayesian reasoning?

Since the earlier observations of Granberg and Brown (1995), several studies have shown an increase in switching rate after several repetitions of the MHD (Friedman, 1998; Granberg and Dorr, 1998; Franco-Watkins et al., 2003; Palacios-Huerta, 2003; Herbranson and Schroeder, 2010; Petrocelli and Harris, 2011; Mazur and Kahlbaugh, 2012; Klein et al., 2013; Saenen et al., 2014). However, in the absence of highly rewarding outcomes (Palacios-Huerta, 2003), a large majority of participants persist in the sub-optimal sticking strategy, switching in none or in only a few trials. As developed below, this impediment can be related to a switching aversion and/or to an *endowment effect* (Kahneman et al., 1991). These emotional influences work against the discovery of the optimal choice by biasing the estimation of the winning probability of the first choice; that is, by inducing an illusion of control (Gilovich et al., 1995; Granberg and Brown, 1995), by biasing the memory of previous choice-outcome events (Petrocelli and Harris, 2011), and/or by preventing the accumulation of enough switching-winning experiences, as shown by a large number of participants in numerous studies.

### Switch Aversion and the Endowment Effect

Similar to findings in other choice contexts (Landman, 1988), studies focusing on the MHD show that people report stronger regret when losing a prize by switching than by sticking (Gilovich et al., 1995; Granberg and Brown, 1995). Interestingly, Petrocelli and Harris (2011) observed that participants overestimated the trials in which they switched and lost, supporting the subjective experience that *switching and losing* is more aversive than *sticking and losing*. An increment of counterfactual thoughts associated with regret after switching and losing seemed to explain this distortion in memory (Petrocelli and Harris, 2011).

Not only do people find switching and losing highly aversive, they also appear to perceive switching and winning as less rewarding than sticking and winning (Franco-Watkins et al., 2003). In one of Franco-Watkins et al. ’s (2003) experiments, participants played several rounds of the MHD after observing the choices and outcomes of a virtual participant in an analogous version of the game. Results showed that the switching rate of the participants was still below 50% even after observing that, in a rigged condition, switching produced 90% of winning trials, whereas the sticking rate was 100% after observing a player sticking and winning 90% of the trials (Franco-Watkins et al., 2003). Accordingly, the *win-stay, lose-switch* strategy shown in other probability learning tasks (e.g., Nowak and Sigmund, 1993) seems to be modulated by the previous choice (sticking or switching) in the MHD.

The switch aversion, or its complementary endowment effect—the tendency to attribute higher value to own options, even when compared to a slight more rewarding alternative (Kahneman et al., 1991)—has also been observed in variations of the MHD which include a larger number of doors (Stibel et al., 2009). That is, the endowment effect has been observed even in conditions where the difference between the final winning probabilities is much higher than in the standard three doors scenario (opening 8 of 9 remaining doors: Franco-Watkins et al., 2003; or opening 98 of 99 remaining doors: Stibel et al., 2009). In the mentioned experiment of Franco-Watkins et al. (2003), participants still preferred sticking with the initial choice even after observing the fictitious participant staying and losing in 90% of the trials (Franco-Watkins et al., 2003; 10C/3D condition). Stibel et al. (2009; Experiments 1 and 4) also found that between 30 and 50% of participants preferred the first choice after opening 98 of 99 remaining doors in one-shot game.

A marked tendency to stick with the first choice has also been observed in a condition in which the second choice was made between the first selection and *both* of the other two options, that is, without the elimination event and, hence, without the need to update probabilities (Morone and Fiore, 2008). As expected, the percentage of participants switching was significantly higher (across 10 trials, the overall switch rate was.58; 8 of 20 of participants had a switch rate higher than.7) compared to the standard MHD (the overall switch rate was.41; only 1 of 20 participants had a switch rate higher than.7). However, the percentage of participants with a switch rate below 0.5 was still not far away from the standard MHD (40 and 50% in “for dummies” and standard versions, respectively; Morone and Fiore, 2008), suggesting that switch aversion or the endowment effect work as attractors toward the non-optimal choice of sticking even in the MHD “for dummies.”

### Overcoming Choice Biases

Granberg and Dorr (1998), Tubau and Alonso (2003), and Stibel et al. (2009) attempted to reduce the endowment effect by eliminating the participants’ first choice. This was accomplished by assigning participants one option among the initial three so that participants only had to choose between sticking and switching. Although the preference for switching was higher than in standard MHD conditions, about 50% of the participants still preferred the first, assigned choice (Tubau and Alonso, 2003). Furthermore, informal reports of the participants showed no improvement in correct Bayesian reasoning, including those participants who switched in most of the trials (Tubau and Alonso, 2003; see also Stibel et al., 2009). Typical comments of participants who finally became aware of the switching advantage believed that the computer program was biased in favor of switching but they expected the same winning probability for both choices (switching and sticking). It could be argued that such conditions hampered the motivation of the participants and, accordingly, their attention to the relevant contingencies was diminished. As observed in other tasks, being able to choose seems to be crucial to engage motivation (Leotti et al., 2010). But in the case of the MHD we have seen that the attraction to the first choice often prevents exploring the consequences of switching, making the discovery of the causes producing the switching advantage even more difficult.

On the other hand, it is well known that the perception of *two* remaining options in the final choice induces the misapplication of the equiprobability principle (Johnson-Laird et al., 1999; Falk and Lann, 2008). Hence, discovering the optimal choice in the MHD can be enhanced by changing the visual appearance of the final choice scenario or by manipulating the number of initial choices. For example, Howard et al. (2007) found higher switching rates in a condition in which all the boxes (closed and open) were visible compared to a condition in which the null options were removed. Increasing the area of the closed boxes also had a significant effect, although smaller than the number-of-boxes manipulation. Hence, the number of visible options seemed to be a relevant factor for promoting switching choices. Evidence of reasoning improvement was not reported but, based on other studies, it seems unlikely that the number-of-boxes manipulation had a significant effect on correct reasoning. In a one-shot scenario, Stibel et al. (2009) showed that, among the participants choosing to switch, probability judgments matched the equiprobability intuition, even in the condition in which 98 of the remaining 99 options were removed! (see also Franco-Watkins et al., 2003).

In addition to the interventions introduced above, increasing incentives (Friedman, 1998; Palacios-Huerta, 2003), or enhancing collaborative playing (Palacios-Huerta, 2003) also seem to be effective for overcoming choice biases in the MHD, at least for some participants. It is worth noting that the most effective intervention appears to be the manipulation of incentives (Palacios-Huerta, 2003), supporting the emotional source of the choice biases observed in the MHD. Unfortunately, none of these latter studies reported probabilistic judgments of the participants. However, based on the results of Stibel et al. (2009), who also used money as a reward, an increment in the amount of gain does not seem to be effective for improving Bayesian reasoning. In the next section we review in more detail the relationship between choice and reasoning improvement in the MHD, as well as possible explanations for the observed dissociation.

### Dissociating Choice from Reasoning

None of the MHD studies assessing the accuracy of probabilistic judgments after several repetitions have observed improvement of correct explicit Bayesian reasoning (Franco-Watkins et al., 2003; Klein et al., 2013; Saenen et al., 2014). In the best case, participants who, following practice, report that switching is more advantageous, tend to switch more often (Tubau and Alonso, 2003), but they are typically unable to explain the reason for that advantage (see also Klein et al., 2013).

It could be argued that the null effect of practice for enhancing understanding the probabilistic structure of the MHD is due to the small amount of practice (commonly less than 50 repetitions). Nevertheless, a larger number of trials appear insufficient for maximizing optimal choice (Herbranson and Schroeder, 2010; Klein et al., 2013; Saenen et al., 2014) or for enhancing correct Bayesian reasoning (Klein et al., 2013; Saenen et al., 2014). For example, after about 250 repetitions of the MHD, only one participant out of 17 seemed to correctly explain the optimal strategy: “First, I clicked on a random box. After one of the boxes disappeared, I clicked on the third box” (Klein et al., 2013), but even this was without clear evidence of having understood the *cause* of the switching advantage. Saenen et al. (2014) analyzed the accuracy of probability judgments in different moments during 100 repetitions of the MHD and found no evidence of improvement at any stage of practice. It is worth noting that Saenen et al. (2014) gave continuous feedback and, in one of the groups, feedback explicitly related winning and losing to each choice (sticking and switching). Although explicit feedback increased frequency of switching, it was not helpful for improving explicit probabilistic judgments.

Accordingly, studies centered on the effect of practice with the MHD suggest that knowledge acquired by learning the different winning probabilities does not lead to better comprehension of the MHD. More specifically, practice seems to facilitate the overcoming of initial choice biases, but does not facilitate an understanding of *why* initial choice tendencies are not optimal. Supporting this claim, significant increments in optimal choice in the MHD have been observed even without explicitly noticing its advantage, although the general tendency to choose optimally (switch) is much weaker than when noticing the switching advantage (Tubau and Alonso, 2003; Klein et al., 2013). In addition to the initial strong bias to avoid switching, these results suggest the involvement of associative mechanisms similar to the ones reported in studies with other non-human animals (Herbranson and Schroeder, 2010; Mazur and Kahlbaugh, 2012; Klein et al., 2013). Associative mechanisms can explain the observed implicit learning of the switching advantage. Nevertheless, without awareness of the rules and effortful control to apply them, they seem to be insufficient to overcome initial choice biases (see Stocco and Fum, 2008, for similar conclusion in other choice tasks).

In line with the associative account introduced to explain the observed dissociation between reasoning and choice, Stibel et al. (2009) concluded that evidential strength, on which choices are based, is sensitive to the evidence provided by alternative hypotheses, but explicit probability judgments are typically less sensitive to slight or apparent changes in support strength (see also Tversky and Koehler, 1994). Accordingly, variables affecting the increment of optimal choice, as for example the increment in the number of non-chosen options, produce an increment in evidence strength for the alternative hypothesis (switch in the MHD) without affecting the corresponding probabilistic judgment (Stibel et al., 2009). Similarly, the effect of repeated practice with the MHD enhances the realization that the proportion of winnings by switching is higher than winnings by sticking, which affects the evidence strength of the final choices. Nevertheless, all these interventions remain insufficient for overcoming the equiprobability illusion, which continues to bias explicit probabilistic judgments.

## Enhancing Probabilistic Reasoning in the MHD

Based on the reviewed evidence, repeated practice and/or higher incentives have a moderate effect on increasing the probability to choose optimally, but it is not useful for enhancing the understanding of the causes of the switching advantage, namely, the prior, conditional, and posterior probabilities involved in the MHD. This section reviews the utility of interventions more directly aimed at improving explicit Bayesian reasoning.

### Explaining Possibilities: Mental Models and the Perspective Effect

The information presented in the text of the problem affects the building of the mental models on which judgments and decisions are based (Legrenzi et al., 1993; Johnson-Laird et al., 1999). In the case of the MHD, different manipulations have been shown to affect reasoning and/or choice. As previously introduced, if instead of the standard dilemma, participants are offered a choice between the selected door and both of the remaining two doors (“for dummies” version in Morone and Fiore, 2008), the tendency to switch increases. It is well documented that decision makers create mental models based on the number of options being presented (Johnson-Laird et al., 1999). If one of the three options is removed, only two models are taken into account: One in which the prize is behind the selected door, and one in which the prize is behind the remaining door (see also Johnson-Laird et al., 1999; Franco-Watkins et al., 2003). Nevertheless, presenting a more transparent MHD does not imply developing a more complete representation, as many individuals have trouble understanding the prior probabilities (Tubau and Alonso, 2003; Tubau, 2008; see below).

The interventions which have been demonstrated to be the most effective for improving correct reasoning in the MHD explicitly request the reasoner to imagine the different possibilities from the different perspectives of both the contestant and the host (Krauss and Wang, 2003; Tubau and Alonso, 2003; Tubau, 2008). For example, using a diagram, Krauss and Wang (2003) presented three closed doors, one representing the selection of a hypothetical contestant. To enhance the representation of the different possibilities from each perspective, participants were asked to imagine being the host of the game who is opening a null door between the two non-selected doors. The percentage of correct justifications of the switching advantage, from the contestant’s point of view, increased from 3% in the standard MHD to 39% in this new presentation (50% correctly noticed the advantage of switching; Krauss and Wang, 2003). Given that participants did not perform the initial choice, it could be argued that the benefit of the intervention was in part a consequence of eliminating the difficulty in overcoming initial choice biases (see Switch Aversion and the Endowment Effect). However, the effectiveness of the perspective manipulation was also observed in an experienced adversary game context, regardless of the role of the participant (Tubau and Alonso, 2003).

More directly, Tubau and Alonso (2003) asked participants to represent the different possibilities from both perspectives. In their third experiment, participants were presented an imaginary card game between two adversaries: The decision maker selecting a card among three (one ace and two other cards), and the informant keeping the other two. Analogous to the host of the MHD, the informant always showed a non-ace card after the decision-maker’s selection. In one experimental condition, participants had to state the possibilities of each player having the ace, and then estimate each player’s likelihood of winning, as well as provide a justification for the perceived best strategy (switching, sticking, or no preference). This condition was compared to the same adversary version, but without the requirement of representing the possibilities, as well as to the standard MHD. Percentage of correct justifications for the switch response were 0% in the standard MHD, 25% in the adversary version without explicit representation, and 60% in the adversary version with explicit representation of possibilities. In sum, encouraging a shift between perspectives seems to be an effective intervention to enhance the building of more complete mental models of the different possible locations of the prize, as well as improved awareness of which options can be eliminated and why. Support for this proposal can also be found in Tor and Bazerman (2003) who, based on protocol analyses in different competitive games, concluded that the main difficulty in competitive contexts is to consider the decisions of others and the rules of the game (the constraints of the host in the MHD).

### Enhancing Correct Probabilistic Judgments: The Role of Natural Frequencies

Another widely discussed facilitator of Bayesian reasoning performance is to present and request problem information as natural frequencies (Gigerenzer and Hoffrage, 1995; Girotto and Gonzalez, 2001; Johnson and Tubau, 2013). Although disagreement persists regarding the specific mechanisms involved in processing natural frequencies (e.g., Gigerenzer, 1994; Barbey and Sloman, 2007), presenting and requesting information in a similar frequency format is also known to facilitate reasoning in the MHD.

**FIGURE 2:**
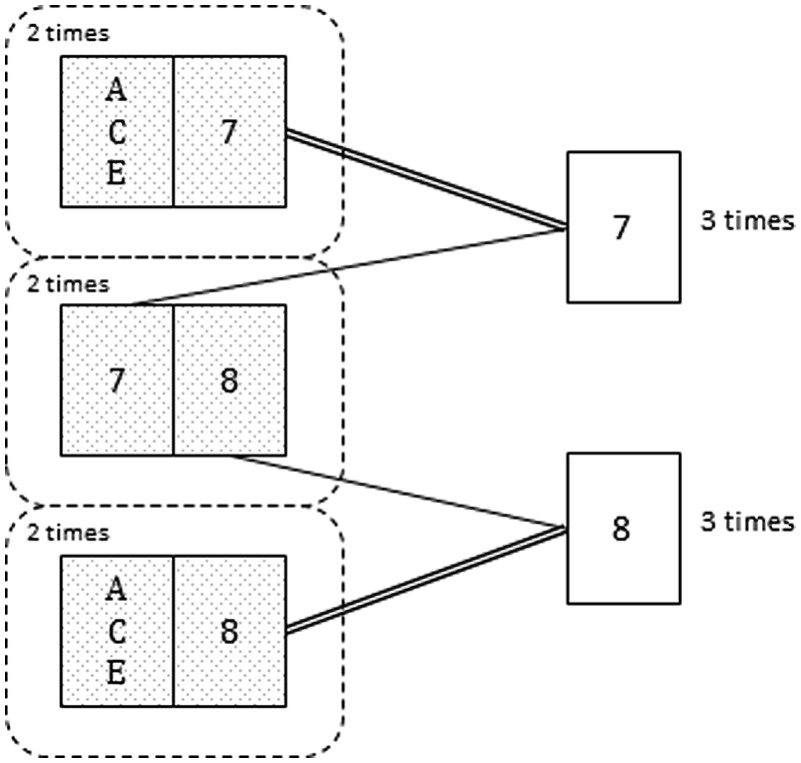
**Card shown by the informant (analogous to the host in the MHD) in six hypothetical repetitions of the game**. Notice that among the three times that the informant shows the 7 (or the 8) he hides the ACE twice (adapted from Tubau, 2008).

For example, Krauss and Wang (2003; Experiment 3) compared the utility of an intervention based on a simplified representation of only three arrangements (similar to first three possibilities in **Table 1**) with a more complete representation of six arrangements (mental model representation from Johnson-Laird et al., 1999; similar to the diagram shown in **Figure 2**, but including the complete representation of each possibility instead of the frequency information). Results showed that the three-arrangements version promoted more correct responses. The benefit of the simplified representation was interpreted as a consequence of its higher resemblance to a natural frequency format (Krauss and Wang, 2003). However, it is not clear which words and numbers were included in the question requiring the probability judgment. As shown in other Bayesian problems, the match between the text of the problem and the text of the question has a significant effect on the responses (Girotto and Gonzalez, 2001; Ayal and Beyth-Marom, 2014). If the question was the same as in Kraus and Wang’s Experiment 2, then there would be a better match between the question (___ out of 3) and the simplified representation (three arrangements) than between the question and the complete version (six models). So, it could be the case that the more complete representation was less effective due to the additional steps needed to transform presented information into the form requested in the question.

Related to the previous hypothesis, in Tubau (2008; Experiments 1A,B) two explanations of an analogous MHD card game were compared: In the *concrete frequency* version, the explanation referred to a specific simulation of six games, analogous to the mental models representation (i.e., in the two cases in which John has the ace and the 7, he will show the 7; in the two cases in which John has the ace and the 8, he will show the 8; and in the two cases in which John has the 7 and the 8, he will show the 7 once and the 8 once; see **Figure 2**). In the *relative frequency* version, less precise verbal quantifiers were used (i.e., if John has the ace and the 7, he will *always* show the 7; if John has the ace and the 8, he will *always* show the 8, and if he has the 7 and the 8 he will show the 7 *half of the times* and the 8 *half of the times*). Each version was presented with and without a diagram similar to the one presented in **Figure 2**. Results showed a significant effect of statistical format (concrete frequencies enhanced performance compared to abstract quantifiers), but no effect was found for the visual diagram. Hence, results supported the Krauss and Wang (2003) and Tubau and Alonso (2003) conclusion regarding the need to build models (possibilities) from both perspectives in a way which facilitates the computation of the respective winning frequencies. As shown in these studies, the highest benefit is observed when participants are externally guided during both the presentation of the problem and via the formulation of the question. Furthermore, and similarly to other Bayesian reasoning problems, the closer the match between the numerical format included in the explanation and the required numerical expression, the higher the benefit (Girotto and Gonzalez, 2001; Ayal and Beyth-Marom, 2014).

### Explaining Causal Relations: Competition Scenarios

According to the studies reviewed so far, probabilistic reasoning in the MHD can be improved through interventions that facilitate building a more complete representation of the different possibilities, or by prompting the required numerical expression in the format of the requested probabilistic judgment (i.e., ___ out of 3). Nevertheless, the extent to which any corresponding improvement indicates a complete understanding of both prior probabilities and the consequences of the elimination’s conditions, (as opposed to simply being a consequence of a match between representations), remains unclear. As developed in Section “Understanding the MHD: From Intuition to Bayesian Reasoning,” understanding the MHD implies understanding that, after the elimination of an option conditioned to the location of the prize, the winning probability of the first choice remains invariant, whereas the winning probability of the remaining option increases twofold.

Related to the comprehension of the elimination’s constraints, a different and interesting approach to improve reasoning in the MHD was developed by Burns and Wieth (2004). Similarly to Glymour (2001), Burns and Wieth (2004) attributed the main cause of failed understanding of the MHD to a failure in understanding the causal structure which produces the switching advantage (see also Krynski and Tenenbaum, 2007, in other Bayesian scenarios). From this perspective, the fact that two independent causes (initial choice and location of the prize) collide on a common effect (the opening of one of the non-selected doors; see **Table 1**) might explain why the MHD is so hard. Based on this assumption, Burns and Wieth (2004) hypothesized that a context more clearly presenting the causes that determine the elimination of an option would enhance reasoning. Supporting this hypothesis, Burns and Wieth (2004) found better performance in analogous MHD competition scenarios (i.e., a competition among three boxers in which only one was the best). However, even in the best conditions of the competition context, only about 50% of the participants selected the optimal (switch) choice and less than 20% of participants were able to express the correct posterior winning probabilities. These results suggest that making more salient the causal conditions that determine the elimination event, or a better knowledge of the rules of the game (Tor and Bazerman, 2003), are also insufficient for a large number of participants to understand the MHD. It is worth noting that clear causal structures seem to primarily benefit higher numerate reasoners in other Bayesian problems (McNair and Feeney, 2014). In the case of the MHD, in addition to the just reviewed difficulties, we suggest that this limitation is also due to a failure in representing the prior probabilities.

## An Overlooked Failure: Incomplete Representation of Prior Probabilities

How people represent the prior probabilities in the MHD has been rarely investigated. In most studies it is assumed that people have an accurate representation of the different probabilities before the elimination event that is, before inducing the equiprobability illusion. However, with the exception of the prior winning probability for the first choice, prior probabilities in the MHD are not necessarily obvious. Representing the winning probability of the initial choice is easy given the transparent correspondence between the initial information, three doors, and one prize, and the correct ratio 1 of 3 chances to win. However, representing the winning probability of the set including the two remaining doors might present a conflict between these two non-selected doors and the three initial doors. In fact, it has been observed that only about 50% of undergraduates understand that the chance of the non-selected options (held by the host or informant in the card game) hiding the ace is 2 of 3, with a common response instead being 1 of 2 (Tubau and Alonso, 2003; Tubau, 2008). Still more difficult is understanding (or expressing) that, among the non-selected options, at least one is null. Only 25% of participants were able to correctly answer the question: “What is the probability that, among the non-selected cards, at least one is not the ace?” (Tubau, 2008). Hence, although most participants are able to represent, in a diagram, the different possible locations of the prize (Tubau and Alonso, 2003), many have difficulties expressing the corresponding probabilities (Tubau and Alonso, 2003; Tubau, 2008).

Weak representation of uncertain information causes vulnerability to biases and/or to conservative behavior (van der Pligt, 1998). Similarly, we argue that one of the consequences of the incomplete comprehension of prior probabilities in the MHD is the vulnerability to the equiprobability illusion. This, together with the choice biases discussed above, promotes the final decision to stick. In particular, susceptibility to the illusion is caused by a weak representation of the facts that: (a) the non-selected doors will hide the prize 2 out of 3 times, (b) among the non-selected doors it is certain that at least one is null, and (c) this null option will always be eliminated. Furthermore, without an adequate representation of the prior probabilities, the perspective manipulation commented above has no effect (e.g., in the adversary version without the explicit representation manipulation in Tubau and Alonso, 2003). Accordingly, being able to understand the elimination’s conditions (the constraints imposed on the host or on the computer), which is crucial for correct Bayesian reasoning in the MHD (Krauss and Wang, 2003; Burns and Wieth, 2004), cannot be useful without an accurate representation of the prior probabilities. It is worth noting that the most effective intervention in Krauss and Wang (2003) was the one prompting reasoners to imagine themselves opening one of the doors according to the elimination’s conditions (perspective effect), together with the requirement to express the answer as a ratio of frequencies: The number of times, out of 3, in which the prize would be behind the contestant’s door. That is, the one promoting the representation of the initial possible locations of the prize.

In sum, a large number of the undergraduates that participate in the MHD experiments do not have adequate knowledge to understand and/or represent prior and conditional probabilities in the MHD (Tubau and Alonso, 2003; Tubau, 2008; see also Brase et al., 2006, for similar claim in the context of other probabilistic reasoning tasks). Therefore, when interpreting the data in the literature, it is important to take into account these limitations. A more complete comprehension of the psychology of the MHD would require the consideration of specific knowledge or skills as mediators of performance.

## Understanding Reasoning Failures in the MHD: A Theoretical Analyses

Although not without critics (for a recent review see Evans and Stanovich, 2013), most current thinking theories share a dual-systems or dual-processing approach. In essence, dual thinking theories consider that effortless, intuitive thinking processes occasionally lead to erroneous or *suboptimal* responses, unless more effortful, analytical reasoning processes intervene to override an initially biased tendency (Evans, 2010; Kahneman, 2011; Stanovich, 2011). Some of the factors that determine the success of effortful reasoning include: Adequate cognitive resources, specific knowledge related to the task, confidence in the intuitive response, and thinking dispositions (engagement or *laziness* of the *reflective* mind). Specifically, Stanovich (2009) suggested that the reasoning system can be understood as including two different “minds”: the algorithmic, which *controls* the running of specific reasoning procedures, and the reflective, which *decides* which reasoning algorithm to apply and/or whether or not to invest more effort into the task. Therefore, according to this proposal, overriding an erroneous response produced by the autonomous mind (Stanovich, 2009) might fail due to lack of resources and/or knowledge to run specific procedures (a failure of the algorithmic mind) and/or due to weak disposition to implement a needed procedure or to review an initial response (a failure of the reflective mind).

Applying this distinction to the MHD, would the frequent but wrong application of the equiprobability principle be a failure of the algorithmic mind? Or would it be consequence of a *lazy* reflective mind? As commented in Section “An Overlooked Failure: Incomplete Representation of Prior Probabilities,” a large number of participants do not have adequate knowledge to correctly represent the prior and conditional probabilities in the MHD (e.g., the probability of the set of non-chosen doors containing the ace; the probability of one of the non-chosen doors being empty; Tubau and Alonso, 2003; Tubau, 2008). For these participants, explicit explanations of the different possibilities during the game had a weak effect on correct reasoning, compared to that observed with higher numerate participants (Tubau, 2008). In addition to a lack of specific knowledge, reasoning in the MHD has been also impaired when the reasoning resources (working memory) were compromised by a secondary task (De Neys and Verschueren, 2006), supporting the relevance of the algorithmic mind for correct reasoning. Nevertheless, it is a common finding that the MHD remains obscure even for high numerate individuals (Girotto and Gonzalez, 2005) or for participants with high working memory span (De Neys and Verschueren, 2006).

Regarding the role of the reflective mind in the MHD, there is no direct evidence of a relation between reflective thinking ability and performance in the MHD. Based on the general finding of strong difficulties in overcoming the equiprobability bias, even for individuals with more education (Girotto and Gonzalez, 2005) or higher working memory span (De Neys and Verschueren, 2006), we anticipate that the relation between reflective thinking capacity and correct reasoning in the MHD would be small or non-existent. It is possible that this relation might emerge if additional relevant information were provided (e.g., explicit representation of the different possibilities), as observed for participants higher in numeracy (Tubau, 2008). But, without this facilitation, weakness of the reflective mind on its own is unlikely to be the main cause of reasoning failures in the MHD.

If people high in cognitive reflection fail to review the erroneous default intuition it may be due to either an absence of the relevant triggering conditions for reflection, or to the absence of adequate knowledge to replace the erroneous default intuition with the correct model of the task (due, for example, to a biased representation of prior probabilities; see Section “An Overlooked Failure: Incomplete Representation of Prior Probabilities”). One of the relevant triggering conditions for reflection is the detection of conflicting beliefs, which tends to reduce confidence in the correctness of the response (Thompson, 2009; De Neys, 2014). In the case of the MHD, experience with the game can produce two different types of conflict: (1) Conflict between correct representation of prior probabilities and the elimination’s conditions and the subsequent equiprobability intuition, and (2) Conflict between the default equiprobable intuition and the experienced switching advantage. None of the reviewed studies have reported confidence measures or other measures of conflict detection. Nevertheless, based on previous findings showing incomplete prior representation and/or the formation of the wrong belief that, after the elimination of an option, a probability update is needed (Baratgin and Politzer, 2010), we anticipate that no conflict (1) would be detected, however, this would be an interesting question to follow up in future studies.

Related to previous conflict (2), there is evidence that noticing it does not improve the chances to override the default intuition (Tubau and Alonso, 2003; Klein et al., 2013; Saenen et al., 2014). For example, in Tubau and Alonso (2003), participants who noticed the conflict between the equiprobability intuition and the switching advantage seemed to solve this contradiction by creating a new explanation (in terms on an anomaly in the computer program). That is, the equiprobability intuition seemed to be accompanied by such a strong feeling of rightness (e.g., Thompson, 2009) that the observation of a discrepancy would have been associated with exception (anomalous program) rather than to a conflict to be solved. Furthermore, if some form of conflict were detected, the biased representation of the underlying probabilistic structure for most participants (see An Overlooked Failure: Incomplete Representation of Prior Probabilities), together with the direct perception of *two* final, *initially equal*, doors would have likely prevented finding the correct solution. In this sense, reasoning failures in the MHD could be attributed to automatic processes which build a particularly *vivid* default mental model of the task, and correspondingly strong justification of its correctness, rather than to a *weakness* of the reflective mind *per se*.

## Implications for Enhancing Bayesian Reasoning

As commented above, participants noticing the switching advantage in the repeated MHD solved the contradiction with the default intuition by building an alternative explanation able to preserve it. This suggests that the *reflective mind* might indeed notice certain conflicting information (conflict 2 in previous section), but the relevant information needed to correct the error in the default intuition (i.e., correct representation of prior probabilities and the elimination’s conditions) is either not available or ignored. Accordingly, the efficacy of interventions aimed at improving Bayesian reasoning in the MHD would depend on the available reasoner skills and/or external hints which enhance the building of a more complete representation of the task. According to the present review, the interventions that have been shown to be the most effective are the ones promoting a different partition of the probability space (Krauss and Wang, 2003; Tubau and Alonso, 2003; Tubau, 2008). Instead of modeling the winning probability of *each of the three options* separately [*P*(each option) = 1/3], understanding the MHD requires modeling the winning probability of each *set of possibilities* corresponding to each actor [i.e., *P*(contestant) = 1/3; *P*(host) = 2/3]. Notice that with this representation, and with the additional knowledge that the host for sure has at least one null option that must be shown, no other computation is needed (see **Table 2**).

**Table 2:** Main beliefs and biases affecting reasoning and choice in the MHD both before and after the elimination of an option.

Incorrect reasoning and choice
Before the elimination of an option	
	Correct application of the equiprobability principle: ***Three equal* options** (frequently, together with incorrect or incomplete representation of the possibilities related to the set including the other two options)
After the elimination of a null option	
	**Reasoning based on cognitive biases** (incorrect comprehension of priors and/or the elimination’s conditions; incorrect application of the equiprobability principle): ***Chances are equal for switch and stick* (1/2 each)**
	**Choice based on emotional biases** (switch aversion; endowment effect; illusion of control): ***Select stick***(consider switching in case of *bizarre* or *unexplainable* observation of switch advantage)

**Correct reasoning and choice**

Before the elimination of an option	
	Correct partition of the probability space: ***Two unequal* sets of possibilities** Chances for the selected option: 1/3 Chances for the other *two* options: 2/3 (and a null option *for sure*)
After the *obligatory* elimination of a null option	
	Correct comprehension of the elimination’s conditions Chances for the selected option: 1/3 Chances for the other option: 2/3 (the null option was *predicted*)
	**Reasoning: *Chances are higher for switch (2/3) than for stick (1/3)*** **Choice: *Switch is a better option than stick***

In sum, as observed in other Bayesian problems, the correct partition of the problem space of probabilities or corresponding set–subset structure is crucial for correct reasoning (Johnson-Laird et al., 1999; Barbey and Sloman, 2007). As also shown in other Bayesian problems, the use of natural frequencies can facilitate the comprehension of the MHD (Krauss and Wang, 2003; Tubau and Alonso, 2003; Tubau, 2008). This seems particularly relevant in case of lower numerate reasoners, who would require a simulation of the partitioned probabilities by simulating several repetitions of the game (Tubau, 2008). But, in general, reviewed findings in the MHD suggest that the accuracy of explicit Bayesian reasoning depends on the accuracy of the underlying partitions of the probability space included in the mental model of the task.

## Conclusion

The strong counterintuitivity of the MHD has intrigued people for decades. What is it about the MHD that makes it so hard for people to know that switching is the best course of action to win the prize? And on top of that, what is it that generates such strong disbelief even if it is realized that switching is better? Assuming the random assignation of the prize, it is clear that, in the initial stage of the game, most people would correctly assign to each alternative the same probability of hiding the prize. It is after the first choice is already made and the second choice to stick or switch is offered that the dilemma develops. The trouble starts with the initially built representation of the task upon which this second decision is based. On the one hand, emotional biases such as anticipation of regret and the endowment effect make people opt for sticking. On the other hand, it has also been suggested that the incomplete representation of the different possible courses of action is normally mediated by ignorance about the constraints involved in the elimination of one option. Nevertheless, as argued in this review, the initial partition between three equally likely options instead of two unequal sets of possibilities (contestant’s and host’s possibilities) seems also to be an important determinant, frequently ignored, for the difficulty in overcoming the equiprobability illusion in the final two-choice scenario.

The relevance of ensuring a correct initial partition of the probability space, combined with understanding that there is a null option within the non-selected partition, is supported by the observation that the best interventions shown to improve Bayesian reasoning in the MHD are the ones promoting the representation of the possibilities of each actor (contestant and host). Furthermore, the dissociation observed between the interventions enhancing optimal choice (repeated practice or increased incentives) and the ones enhancing correct reasoning (explicit partitioning of possibilities) is coherent with current dual process theories of thinking (e.g., Sloman, 1996; Evans, 2010; Kahneman, 2011; Stanovich, 2011) and with dual process models of reward learning (Dayan and Daw, 2008). Whereas changes in preference would be controlled by the autonomous mind (i.e., by means of model-free reward learning mechanisms), explicit reasoning would depend on available cognitive resources and explicit knowledge of the task (similarly to the requirements of model-based reward mechanisms). Accordingly, the present review highlights promising new avenues to help understand behavior and reasoning gaps, and to anticipate the efficacy of new interventions to improve Bayesian reasoning.

## Conflict of Interest Statement

The authors declare that the research was conducted in the absence of any commercial or financial relationships that could be construed as a potential conflict of interest.
